# Chylothorax after chimeric antigen receptor T cell therapy for relapsed and refractory diffuse large B-cell lymphoma: A case report

**DOI:** 10.1097/MD.0000000000035432

**Published:** 2023-10-13

**Authors:** Hsin-Hui Chen, Cheng-Yi Kuo, Ching-Liang Ho, Yeu-Chin Chen

**Affiliations:** a Department of Internal Medicine, Tri-Service General Hospital, National Defense Medical Center, Taipei City, Taiwan; b Department and Graduate Institute of Biology and Anatomy, National Defense Medical Center, Taipei City, Taiwan; c UWELL Biopharma Inc., New Taipei City, Taiwan; d Division of Hematology & Oncology, Department of Internal Medicine, Tri-Service General Hospital, National Defense Medical Center, Taipei City, Taiwan.

**Keywords:** CAR-T, case report, chylothorax, cytokine release syndrome, diffuse large B-cell lymphoma

## Abstract

**Rationale:**

Anti-CD19-targeted chimeric antigen receptor (CAR) T cell therapy is effective in treating relapsed/refractory diffuse large B-cell lymphoma (DLBCL). This therapy is associated with several side effects that can be life-threatening such as cytokine release syndrome (CRS). However, chylothorax associated with CRS after CAR-T therapy has not been reported.

**Patient concerns:**

A 23-year-old male diagnosed with DLBCL relapsing after autologous peripheral blood stem cell transplantation was treated with anti-CD19-targeted CAR-T cell therapy. After CAR-T cell transfusion, he developed grade 3 CRS includes fever, dyspnea, tachycardia and hypotension. The symptoms of CRS persisted and chest plain film revealed bilateral pleural effusion.

**Diagnosis:**

Chylothorax was confirmed by the pleural effusion analysis that triglyceride level was 1061 mg/dL. Bacterial and fungal culture of pleural fluid reported no pathogen was detected. Cytological examination of pleural effusion revealed no malignant cells.

**Interventions:**

The chylothorax resolved after treatment with intravenous administration of tocilizumab.

**Outcomes:**

On 30-day follow-up, the patient was in stable clinical condition with complete remission of DLBCL on whole-body positron emission tomography scan.

**Lessons:**

We reported a rare case of CAR-T associated chylothorax in a patient with relapsed and refractory DLBCL. Grade 3 CRS with high interleukin-6 level was presented in our patient. The symptoms of CRS were improved with tocilizumab treatment and the chylothorax resolved later on. It is suggested that high interleukin-6 releases might induce chyle leakage resulting from activations of endothelium and coagulation. Our finding highlights the occurrence of chylothorax during the course of CAR-T cell therapy and the importance of proper monitoring and prompt management of this life-threatening side effect.

## 1. Introduction

Chimeric antigen receptor (CAR) T cells are genetically reprogramed T cells expressing recombinant receptors against targeted tumor antigen. Anti-CD19 CAR-T cell therapy had proved exciting outcomes in treating acute lymphoblastic leukemia as well as B cell lymphoma and the food and drug administration approved 2 CAR-T cell products in 2017.^[1]^ Anti-CD19-targeted CAR-T cell therapies can achieve a complete remission rate of approximately 50% in patients with relapsed/refractory diffuse large B-cell lymphoma (DLBCL).^[2,3]^ In spite of excellent clinical results, anti-CD19-targeted CAR-T cell therapy is associated with some side effects such as cytokine release syndrome (CRS) which can be life-threatening.^[4]^ The common manifestations of CAR-T-related CRS include fever, dyspnea, tachycardia, hypoxia and hypotension. In addition to CRS, immune effector cell-associated neurotoxicity syndrome (ICANS), infections and cytopenia are other frequent complications associated with CAR-T cell therapy. In this report, we present a patient with relapsed and refractory DLBCL who received CAR-T cell therapy and complicated with a chylothorax.

## 2. Case presentation

A 23-year-old male patient has no history of systemic disease nor cancer-related family history. He complained of abdominal cramping pain and presented to the emergency department at another hospital in July, 2020, where he was diagnosed with intussusception and admitted for right hemicolectomy. During hemicolectomy, abnormal lymph nodes were found and subsequent abdominal ultrasonography revealed multiple hepatic nodules. A liver biopsy was performed for pathological examination and a diagnosis of B-cell lymphoma, stage IV (non-germinal center type, positive for CD20, bcl2, bcl6 and Mum-1; negative for CD3, CD5, CD10 and EBER; C-Myc 30%, Ki-67 80%) was made. He had undergone 6 courses of dose-adjusted EPOCH-R (etoposide, prednisone, vincristine, cyclophosphamide, doxorubicin, and rituximab) and partial response was achieved. He then received autologous peripheral blood stem cell transplantation with conditioning chemotherapy of BEAM regimen (BCNU, etoposide, Ara-C, and melphalan) as consolidation treatment setting. Residual abdominal tumors were irradiated after autologous peripheral blood stem cell transplantation. However, severe abdominal pain and obvious abdominal distension occurred in January, 2022. After relapsed tumors assessed by whole-body positron emission tomography (PET) scan, he received another 6 times of radiotherapy for symptoms relief as palliative treatment. The patient was referred to our institution in January, 2022 and recruited into a CAR-T clinical trial for relapsed/refractory B-cell non-Hodgkin lymphoma (ClinicalTrials.gov: NCT04296461).

**Figure 1.**
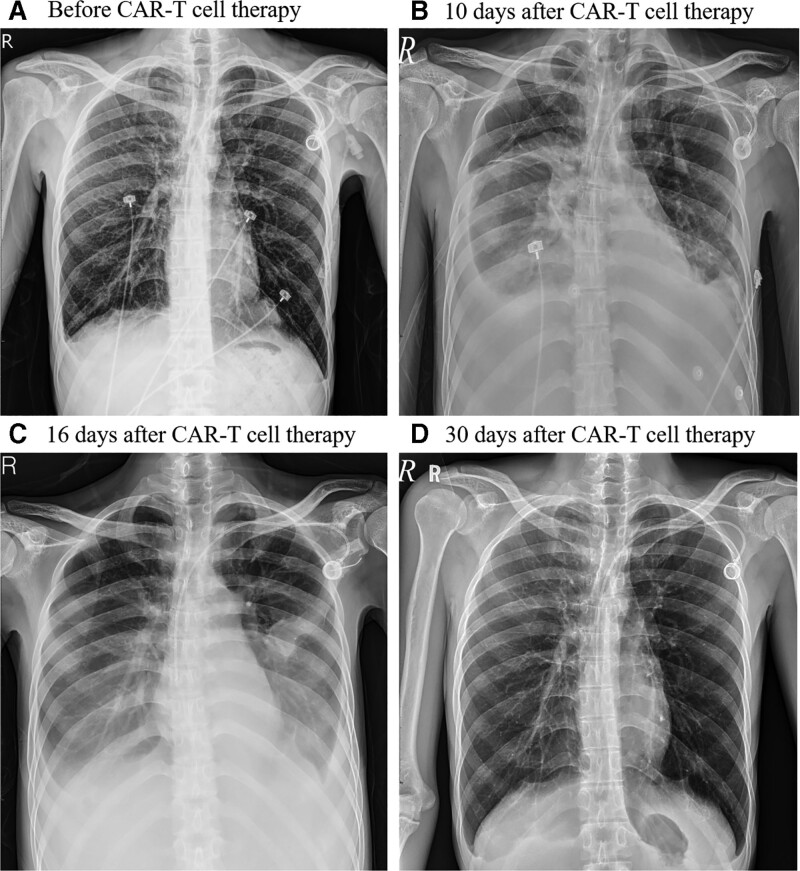
Serial chest plain films before and after CAR-T therapy. CAR = chimeric antigen receptor.

**Figure 2.**
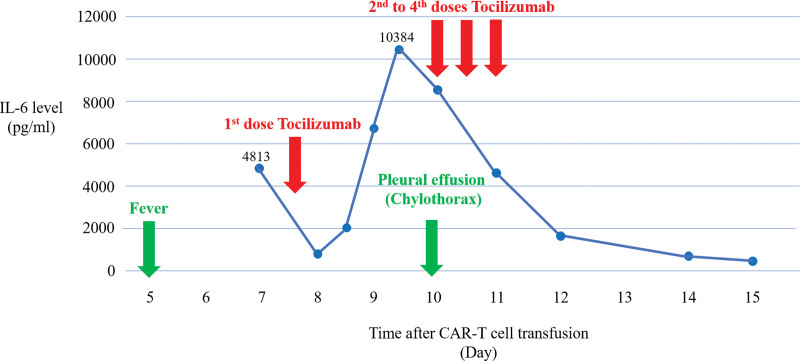
Time course of IL-6 levels, tocilizumab injection and clinical condition. IL = interleukin.

**Figure 3.**
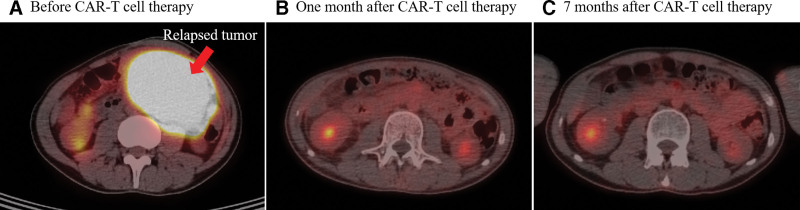
Whole-body PET scan before and after CAR-T therapy. PET = positron emission tomography, CAR = chimeric antigen receptor.

The patient received lymphodepleting chemotherapy with fludarabine 30 mg/m^2^ and cyclophosphamide 300 mg/m^2^ on day −4, −3, and −2 from the manufactured CAR-T cell transfusion. A total of 1.9 × 10^8^ CAR-T cells were administrated in a split-dose schedule on 3 consecutive days with 10%, 30%, and 60%. He developed fatigue, diarrhea and high fever of 39°C on day 5. A serial of examinations disclosed no obvious infectious source, the patient was given with prophylactic antibiotics due to severe neutropenia. The patient experienced tachycardia (120 beats per minute), dyspnea (20–24 breaths per minute) and hypotension (systolic blood pressure around 90–100 mm Hg). A vasopressin with norepinephrine 0.216 to 0.324 μg/kg/minute was prescribed for the treatment. High interleukin (IL)-6 level of 4813 pg/mL was noted on day 7. Based on the clinical symptoms and laboratory finding, the patient was diagnosed with grade 3 CRS and was given with intravenous administration of tocilizumab 8mg/kg for treatment on day 8. However, high fever of up to 40°C, tachycardia around 150 beats per minute and rapid respiratory rate around 30 to 40 breaths per minute were persistent and exaggerated. Physical examination disclosed progressive distension of abdomen and edema of bilateral lower limbs. Abdominal sonography showed moderate ascites. In addition, decreased breathing sound of right side was observed on day 10. Chest plain film revealed bilateral pleural effusion especially the right chest (Fig. 1B) which was not found in admission survey (Fig. 1A). Diagnostic and therapeutical thoracentesis of right side was performed. Dyspnea was relieved after the thoracocentesis. Chylothorax was confirmed with the presence of 98% of mononuclear cells and triglyceride level of 1061 mg/dL by the pleural effusion analysis (Table 1). Bacterial and fungal culture of pleural fluid reported no pathogen was detected. Cytological examination of pleural effusion revealed no malignant cells. The patient was given with 3 consecutive doses of intravenous tocilizumab 8 mg/kg every 8 hours on day 10 due to persistently severe CRS and surged IL-6 level (Fig. 2). The treatment led to much improvement of symptoms in terms of high fever, tachycardia, dyspnea and hypotension. The distension of abdomen and the edema of lower limbs were improved as well. Chest plain film for follow-up on day 16 showed decreased amount of bilateral pleural effusion (Fig. 1C). The patient has no abdominal pain nor other discomfort after the treatment. He was discharged with stable conditions 24 days after CAR-T cell transfusion. On 30-day follow-up, the patient was in stable clinical condition with resolved pleural effusion of bilateral pleural space on chest plain film (Fig. 1D) and sustained complete remission of DLBCL assessed by whole-body PET scan at 1 month and 7 months after CAR-T cell therapy (Fig. 3).

**Table 1:** Laboratory tests of pleural fluid analysis.

Lab test	Value
Pleural fluid examination	
Total cell count	<3104/µL
RBC count	<3000/µL
WBC count	104/µL
PMN%	2%
MN%	98%
pH fluid	7.71
Biochemistry examination of pleural fluid	
Glucose	116 mg/dL
Total protein	2.2 g/dL
Albumin	1.8 g/dL
Triglyceride	1061 mg/dL
Cholesterol	86 mg/dL
LDH	502 U/L
Amylase	18 U/L
Lipase	38 U/L

## 3. Discussion

We here report a rare case of CAR-T associated chylothorax in a patient with relapsed and refractory DLBCL. The chylothorax developed accompanying with CRS 10 days after CAR-T cell transfusion. The patient’s medical history and some workups suggested no definite etiology such as underlying lymphoma involvement. Patient’s DLBCL responded to CAR-T cell therapy well with achievement of complete remission.

Immunotherapy with CAR-T cells targeting CD19 has demonstrated great clinical success in hematologic malignancies.^[5,6]^ Common toxic effects associated with CAR-T cell therapy include CRS and ICANS. These adverse effects are attributed to the unique nature of CAR-T cells engaging designated tumor antigens. The engagement activates the proliferation of CAR-T cells, resulting in eradication of tumor. The tumor cell eradicated by activated CAR-T cells lead to the first wave of proinflammatory cytokine production including GM-CSF, IL-1β, IFN-γ, and TNF-α.^[7]^ The elevated cytokines production in turn triggers another wave of cytokines including IL-6, IL-8, and IL-10, which originate from bystander myeloid cells such as macrophages and nonimmune cells like vascular endothelial cells. The cytokines produced in the 2 waves generate a positive-feedback loop that intensifies the systemic inflammatory responses contributing to the severity of CRS after CAR-T transfusion. IL-6 is considered to play a central role in pathogenesis of CRS, which is rapidly produced by immune cells such as monocytes and nonimmune cell like endothelial cells and fibroblasts in response to CAR-T cell activation. Circulating level of IL-6 rises within 3 to 10 days after the transfusion of CAR-T cells and declines along with tumor regression 10 to 21 days post-infusion.^[8,9]^ Serum level of IL-6 has been suggested to correlate with the severity of CRS in patients treated with CAR-T cells.^[10,11]^ Blocking IL-6 signaling has been demonstrated to effectively mitigate clinical symptoms of CRS induced by CAR-T cell therapy.^[12,13]^ In our patient, CRS developed on day 5, followed by a significant release of IL-6 on day 7 post-infusion. Despite of a transient decrease of IL-6 level treated with tocilizumab, progression of CRS was observed with a much higher level of IL-6 as shown in Figure 2. The pattern of IL-6 release in the course of CRS is consistent with previous reports showing a peak of IL-6 rising at 7^th^ day after CAR-T transfusion.^[8,14]^ The patient’s clinical status was deteriorated with increasing IL-6 release and complicated by the consequent occurrence of chylothorax. Given no preexisting chylothorax before CAR-T transfusion, the chyle leakage is speculated to be correlated with high IL-6 release that induced endothelial activation and coagulation activation. Elevated endothelial activation biomarkers such as von Willebrand factor and angiopoietin-2 were observed in patients with severe CRS in association with endotheliopathy and coagulopathy.^[15–17]^ Elevated IL-6 production increased vascular endothelial permeability through VE-cadherin disassembly and C5a receptor expression, leading to vascular leakage.^[17,18]^ Lymphatic vessels share structural similarity with blood vessels. The endothelial cells lining lymphatic vessels have been shown to increase the permeability in response to proinflammatory cytokines such as IL-6, which are similar to those observed in the vascular endothelial cells.^[19]^ As the chyle leakage occurred after the second peak of IL-6 production, our patient’s chylothorax is suggested to be attributed in part to IL-6 inducing lymphatic leakage.

Chylothorax can be divided etiologically into 2 categories including traumatic and nontraumatic causes. Causes of traumatic chylothorax are classified as iatrogenic such as injuries during thoracic surgeries or non-iatrogenic like lymph duct damage resulted from trauma or fractures. The etiologies of nontraumatic chylothorax include hematologic malignancy, sarcoidosis, amyloidosis, and lymph vessels disorder.^[20,21]^ Chylothorax are conventionally treated with radiologic intervention and surgical modalities. In cases of drug-induced chylothorax, symptoms were resolved by discontinuing the medication.^[22,23]^ In our case, a huge intra-abdominal mass was noted on PET scan prior to CAR-T cell therapy and there was no chylothorax or pleural effusion observed initially. After CAR-T cell therapy, a chest plain film revealed pleural effusion bilateral and abdominal sonography showed moderate ascites. Lower limbs edema was also found as well. Chylothorax was confirmed by the diagnostic thoracentesis and laboratory analysis. Our patient presented grade 3 CRS with extremely high level of IL-6. The symptoms of CRS were improved with repeated treatment of tocilizumab and the chylothorax was resolved. We speculate that elevated levels of IL-6 during CRS positively correlated with the increased permeability of lymphatic vessels that led to a chyle leakage. Resolution of chylothorax suggests that the influence of IL-6 on the endothelial cell-to cell junction could be managed successfully.

We present a rare case of chylothorax development during severe CRS period after CAR-T cell therapy for relapsed and refractory DLBCL. While clinicians have been aware of 2 common side effects of CAR-T cell therapy, CRS and ICANS, the case reported here highlights the CRS-related other toxicity that may have impacts on morbidity or mortality. The safety of CAR-T cell therapy is highly dependent on effective monitoring and prompt management of toxicities.

## Acknowledgments

We thank all the clinical staffs for taking care of the patient.

## Author contributions

**Writing – original draft:** Hsin-Hui Chen, Cheng-Yi Kuo.

**Writing – review & editing:** Hsin-Hui Chen, Ching-Liang Ho, Yeu-Chin Chen.
